# In the Treatment of Choleraic Diarrhœa

**Published:** 1893-09

**Authors:** 


A practitioner devoting especial attention to the diseases of children, says:

"In the treatment of Choleraic Diarrhœa we are safe, it matters not at what time we may be called, in administering some antiseptic medication, something which will prevent fermentation, and have a destructive effect upon the septic germs more than likely present in the alimentary canal. Happy effects are often secured by the use of Listerine properly diluted; a favorite prescription is the following:

℞ Lambert's Listerine.
Glycerine (c. p.)
Syr. Simpl.
Aquæ cinnamon, aa ℥j. M.

Sig. Teaspoonful every one, two or three hours, as may be indicated.

Taking into consideration the component parts of Listerine, it impresses me favorably as a prophylactic and remedial agent for cholera, along with other intestinal disturbances. The eucalyptus, thyme, gaultheria and boraric acid which it contains are all antagonistic to germ life and oppose fermentation. The preliminary diarrhœa (cholerine, as it is called) may well receive teaspoonful doses of Listerine combined with the same amount of glycerine; in fact, I should be inclined to recommend to the laity this combination as a prophylactic measure."

